# Harnessing the Power of Large Language Models (LLMs) for Electronic Health Records (EHRs) Optimization

**DOI:** 10.7759/cureus.42634

**Published:** 2023-07-29

**Authors:** Abdulqadir J Nashwan, Ahmad A AbuJaber

**Affiliations:** 1 Nursing, Hamad Medical Corporation, Doha, QAT

**Keywords:** artificial intelligence, clinical decision-making, gpt-4, chatgpt, large language models, electronic health records

## Abstract

This editorial discusses the potential benefits of integrating large language models (LLMs), such as GPT-4, into electronic health records (EHRs) to optimize patient care, improve clinical decision-making, and promote efficient healthcare management. Artificial intelligence (AI)-driven LLMs can revolutionize healthcare practices by streamlining the data input process, expediting information extraction from unstructured narratives, and facilitating personalized patient communication. However, concerns related to patient privacy, data security, and potential biases must be addressed to ensure equitable healthcare for all. Therefore, we encourage healthcare professionals and researchers to explore innovative solutions that leverage AI capabilities while addressing the challenges associated with privacy and equity.

## Editorial

In recent years, a special interest has developed among professionals in the field of health informatics for the implementation of advanced artificial intelligence (AI) technologies, particularly large language models (LLMs), within electronic health records (EHRs). These experts assert the belief that leveraging AI in this manner can profoundly enhance the functionality of EHRs, resulting in optimized patient care, improved clinical decision-making, and more efficient overall healthcare management.

This interest has been bolstered by recent breakthroughs in the fields of natural language processing (NLP) and machine learning (ML), which have created new opportunities for innovation in healthcare. For instance, LLMs have demonstrated exceptional capability in comprehending and processing complex language structures [1]. When integrated into EHRs, these AI models can simplify and automate many complex tasks, leading to enhanced productivity and improved healthcare outcomes.

In line with this, several high-profile collaborations between tech giants and healthcare companies are underway. Notably, Microsoft Corp. and Epic have announced an expanded partnership to integrate generative AI, a subtype of AI that can generate new, novel outputs, into healthcare. As part of this initiative, they will be leveraging the Azure OpenAI Service in conjunction with Epic's EHR software [2]. The ultimate goal of this collaboration is to provide AI-powered solutions that can increase productivity, enhance patient care, and bolster the financial integrity of health systems worldwide.

Some of the initial solutions being developed include auto-drafting message responses for prominent health institutions like UC San Diego Health, UW Health, and Stanford Health Care. Another feature under development involves natural language queries in Epic's self-service reporting tool, SlicerDicer. This partnership builds on the history of collaboration between Microsoft, Nuance, and Epic, which has been aimed at enabling healthcare organizations to benefit from Microsoft Cloud and Epic technologies [2].

Incorporating AI-powered language models can significantly enhance the data input process within EHRs. These AI models enable precise and efficient transcription of spoken language, thus streamlining the often time-consuming process of documenting patient interactions [3]. For instance, clinicians could dictate their notes, which would then be transcribed in real-time by the AI models, thereby reducing the need for manual documentation. These models can also help extract important information from unstructured clinical narratives, using NLP techniques to identify and summarize crucial clinical information from patient records. As a result, healthcare providers can access pertinent information more efficiently and effectively, enhancing clinical decision-making.

The use of LLMs can also facilitate personalized patient communication and education. Given their deep understanding of human language, these models can generate tailored, comprehensible, and empathetic patient communication [4]. When integrated within EHRs, healthcare providers can leverage these capabilities to develop personalized care plans and ensure patients are well-informed and actively engaged in their treatment processes.

However, while integrating LLMs into EHRs holds significant promise, it also raises some important ethical and legal concerns, particularly concerning patient privacy and data security. It is essential to ensure that these models comply with stringent privacy regulations, such as the Health Insurance Portability and Accountability Act (HIPAA) in the United States and the General Data Protection Regulation (GDPR) in the European Union [5]. ML models should also be trained and validated on diverse, representative datasets to ensure the accuracy and fairness of their predictions. To address the interpretability problem, the AI system should provide explanations for its predictions or recommendations, which will help healthcare providers make informed decisions. Moreover, patient autonomy (through patient-centered care approach) and informed consenting are vital aspects of healthcare, ensuring decisions reflect patients' preferences and values. The use of LLMs in healthcare should respect these principles and fully inform patients about AI involvement. To democratize LLMs, it is essential to create accessible and user-friendly interfaces and to educate the public about AI, facilitating broader understanding and engagement. Additionally, ongoing research and development efforts are needed to minimize biases in these AI models and promote fairness so that the resulting healthcare solutions are equitable for all.

In conclusion, the integration of LLMs into EHRs represents an exciting development with the potential to revolutionize healthcare delivery. By leveraging these AI-driven models, clinical efficiency, decision-making, and patient communication can be significantly enhanced. As such, researchers and professionals in the field of health informatics should focus on exploring and developing innovative solutions that harness the power of these models while simultaneously addressing critical issues related to privacy and fairness.
